# Optimum Preparation Conditions for Highly Individualized Chitin Nanofibers Using Ultrasonic Generator

**DOI:** 10.3390/polym13152501

**Published:** 2021-07-29

**Authors:** Dagmawi Abebe Zewude, Hironori Izawa, Shinsuke Ifuku

**Affiliations:** 1 Graduate School of Engineering, Tottori University, 4-101 Koyama-Minami, Tottori 680-8550, Japan; d19t3106x@edu.tottori-u.ac.jp (D.A.Z.); h-izawa@tottori-u.ac.jp (H.I.); 2 Center for Research on Green Sustainable Chemistry, Tottori University, Tottori 680-8550, Japan

**Keywords:** partial deacetylation, nanofiber, ultrasonic treatment

## Abstract

α-Chitin derived from crab shells was treated with 30% sodium hydroxide to prepare partially deacetylated chitin with a deacetylation degree of 36%. Partially deacetylated chitin nanofibers were prepared by applying weak ultrasonic energy generated by a domestic ultrasonic cleaner. The deacetylated chitin was easily disintegrated into nanofibers with the aid of electrostatic repulsion and osmotic pressure effect of amino cations on the chitin surfaces. The nanofibers were characterized in terms of yield, morphology, crystallinity, viscosity, and dispersibility. After a series of characterizations, ultrasonication with 45 kHz frequency and 20 min treatment was found to be the optimum conditions for obtaining fine nanofibers with a high yield.

## 1. Introduction

Chitin is the main component of crab and shrimp shells. It has a chemical structure similar to cellulose since it is a repeating structure of 2-acetamido-2-deoxy-*β*-D- glucopyranose. The annual synthetic amount is estimated to be 10^10^–10^11^ tons, which is the second largest resource after cellulose [1]. However, most of the crab shells are discarded without effective utilization. This is due to the fact that chitin is difficult to process and commercialize on account of its low solubility and dispersibility in water and common solvents.

We have produced nano-chitin by mechanical crushing of chitin extracted from crab shells by utilizing the preparing technology of nanocellulose [2]. All-natural chitin is composed of nano-sized crystalline fibrils [3] and can be converted to nanochitin by downsizing with a strong mechanical load. Nanochitin can also be produced from shrimp [4] and mushrooms [5]. Nanochitin is a fibrous substance with a width of 10–20 nm. Since it is a highly crystalline fiber, it has excellent physical properties [6]. It can be molded into a desired shape depending on the application due to its uniformly water-dispersible characteristics [7,8]. Moreover, we have elucidated various biological functions of nanochitin and its partially deacetylated derivatives. For example, effects on the skin, a wound healing effect [9], a relief effect of dermatitis [10], and a hair growth effect [11] have been clarified. As an effect associated with taking the nanochitin, a relief effect of inflammation on the intestinal tract [12] and anti-hepatic and antioxidative effects [13] have been demonstrated. By utilizing various functions, various commercial products containing nanochitin as a functional ingredient have been available such as cosmetics, skin care products, and healthy foods.

Powerful crushing equipment such as grinder and high-pressure homogenizer are often used to downsize chitin. The former equipment crushes chitin by the strong shearing force generated from two rotating grindstones [2]. The latter equipment crushes chitin by colliding a sample ejected at a high pressure from a nozzle [14]. These powerful equipments are needed to break the strong hydrogen bonds between chitin fibrils.

As a derivative of nanochitin, there is a partially deacetylated chitin nanowhisker [15]. This derivative is mostly deacetylated on the chitin surface by sodium hydroxide. The amino group at the C2 position on the partially hydrolyzed chitin is densely cationically charged under acidic conditions. Its cationic property brings electrostatic repulsion and osmotic effects, allowing nanowhiskers individualization and stable dispersion.

There is an ultrasonic generator as a kind of crushing device. Ultrasound initiates compression and rarefaction cycle. During the rarefaction, micro vacuum bubbles are created at various nucleation site, which then implode in the next high-pressure phase. It is a continuous formation of growth and implosion with a release of energy stored within the vacuum bubble [16]. The collapsing bubble creates a mechanical impact on the suspended chitin in the form of shock energy and length wise stress [17]. If the impact energy exceeds the force that binds the nanochitin together, they can break down in the form of nanofibers. The efficiency of this process is strongly influenced by ultrasonication frequency and particle size [18,19].

In this study, an ultrasonic generator with a variable frequency was applied to prepare partially deacetylated nanochitin. The equipment applied is commonly used for cleaning glassware with low power. The cationic charge on the surface of the chitin fibrils may allow for the disintegration of chitin by the cheap ultrasonic generator. The optimum ultrasonic frequency and treatment time were determined from the results of yield, shape, and physical and chemical properties to obtain the partially deacetylated nanochitin effectively. The production of nanochitin using an ultrasonic generator has already been reported [20]. However, in previous studies, chitin without deacetylation was treated with a high-power device for cell disruption at a constant frequency. The mechanical treatment using an ultrasonic generator with low power is characterized by its low acquisition cost, low energy consumption, and by being contamination-free. If partially deacetylated nanochitin are easily prepared by using it, the practical uses of chitin as a functional ingredient may increase.

## 2. Materials and Methods

### 2.1. Materials

Chitin powder from crab shell was purchased from Koyo Chemicals Industry Co., Ltd. (Hyogo, Japan). The degree of deacetylation was 6%. Sodium hydroxide and acetic acid were purchased from Wako Pure Chemical Industries, Ltd. (Osaka, Japan). and used as received.

### 2.2. Partial Deacetylation of Chitin

Partially deacetylated chitin was prepared referring to the previous report with slight modifications [15]. In the process, α-chitin (45 g) was suspended in a 30 wt% NaOH aqueous solution (1500 g) and the suspension was heated at 90 °C for 12 h with continuous stirring. After deacetylation, the precipitate was collected and washed repeatedly by a centrifuge until the supernatant became neutral with the rotational speed of 10,000 rpm for 10 min. A portion of the obtained product was kept for determination of degree of deacetylation and the rest was dispersed in a 1 wt% acetic acid aqueous solution. The degree of deacetylation of the partially deacetylated chitin was determined by the electroconductivity titration method [15]. 0.1 mol L^−1^ HCl was added to the chitin slurry to adjust the pH to 2.8, and 0.1 mol L^−1^ NaOH solution was added at 0.3 flow rate of mL min^−1^ up to pH 11. The conductivity and pH curves reflected the degree of deacetylation of chitin.

### 2.3. Ultrasonic Treatment of Partially Deacetylated Chitin

Partially deacetylated α-chitin slurry was stirred for six days for agitation followed by adjusting the solid content to 0.55 wt% using 1 wt% acetic acid. The obtained suspension, 200 g for each run, was transferred into 500 mL glass beaker and stirred for 30 min under a vacuum to remove air bubbles. Then, the suspension was subjected to ultrasonication process, using three frequency ultrasonic cleaner (VS-100 III, AS ONE, Osaka, Japan) in water bath under stirring. Ultrasonic frequencies were 28, 45, and 100 kHz, and sonication times were 5, 10, 15, 20, 25, and 30 min. After sonication, large chitin fibers were removed by centrifuging. The yield of partially deacetylated chitin nanofibers dispersed in the supernatant were gravimetrically calculated.

### 2.4. Characterizations of Partially Deacetylated Chitin Nanofibers

Field emission scanning electron microscope (FE-SEM, JSM-6700F, JEOL, Tokyo, Japan) was used to ascertain the morphology of different partially deacetylated chitin nanofibers. Cast film was prepared and coated with an approximately 2 nm Pt layer by using an ion sputter coater and observed at 2.0 kV.

Atomic force microscopy (AFM, Nanocute, SII Instruments, Chiba, Japan) was used to measure the width of nanofibers. Diluted chitin nanofiber dispersion was dropped on freshly cleaved mica substrate, dried at room temperature, and subjected to AFM observation. The widths of chitin nanofibers were measured by selecting the measuring points along the fiber axes.

Particle sizes and zeta-potentials of the partially deacetylated chitin nanofibers dispersed in water were measured using dynamic light scattering method (ELSZ-1000ZS, Otsuka Electronics, Osaka, Japan). The concentration of the dispersion for particle size and zeta potential measurement was 0.01 wt% and 0.005 wt%, respectively, at a pH of 3.

X-ray diffraction profile of the chitin nanofiber was obtained in the range of 5° ≤ 2*θ* ≤ 35° by using Ni-filtered CuK*α* from an X-ray generator (Ultima IV, Tokyo, Rigaku) operating at 40 kV and 30 mA. The dry sample was prepared by freeze drying and then pressing into flat sheet at pressure of 20 MPa to generate the profile. Crystallinity index (CI) was determined based on the equation: CI = (*I*_110_ − *I*_am_) × 100/*I*_110_, where *I*_110_ is the maximum intensity of the 110 plane and *I*_am_ is the intensity of the amorphous diffraction at 16° [21].

Viscosity of the partially deacetylated chitin nanofiber suspension was measured by a Brookfield digital viscometer DV-E using spindle no. LV-4 (Brookfield Engineering Laboratories, Middleboro, MA, USA) after raising the solid content to 0.60 wt% by using a rotary evaporator.

The light transmittance of partially deacetylated chitin nanofiber dispersion with 0.1 wt% was measured using a UV-Vis spectrophotometer (V550; JASCO, Tokyo, Japan). The spectra were recorded in the range of 200 to 800 nm.

## 3. Results and Discussion

### 3.1. Ultrasonic Treatment of Partially Deacetylated Chitin with Different Frequency

Deacetylation of chitin with 30 wt% NaOH at 90 °C for 12 h partially converts acetamide groups to amino groups. The degree of deacetylation was 36% as determined by conductometric titration. This value is reasonable compared to previous studies [15]. The amino group of partially deacetylated chitin is protonated under acidic conditions (–NH_3_^+^). Positively charged chitin easily swells by electrostatic repulsive force and osmotic effect, resulting in facilitation of ultrasonication. Partially deacetylated chitin was treated with an ultrasonic generator. The frequencies are 28, 45, 100 kHz. At frequencies of 28 and 45 kHz, the partially deacetylated chitin became a clear dispersion. This suggests that chitin was disintegrated to the nano level by ultrasonic waves, and the nano-sized chitin was individualized and dispersed in water. The ultrasonic generator brings ultrasonic vibrations to the water. The pressure difference due to vibration generates fine bubbles, which gives an impact to chitin and crushes it. During the ultrasonic treatment, low speed mechanical stirring of the chitin suspension is important. The efficiency and reproducibility of the ultrasonic wave impact on the chitin is improved by stirring at 65 rpm.

**Figure 1:**
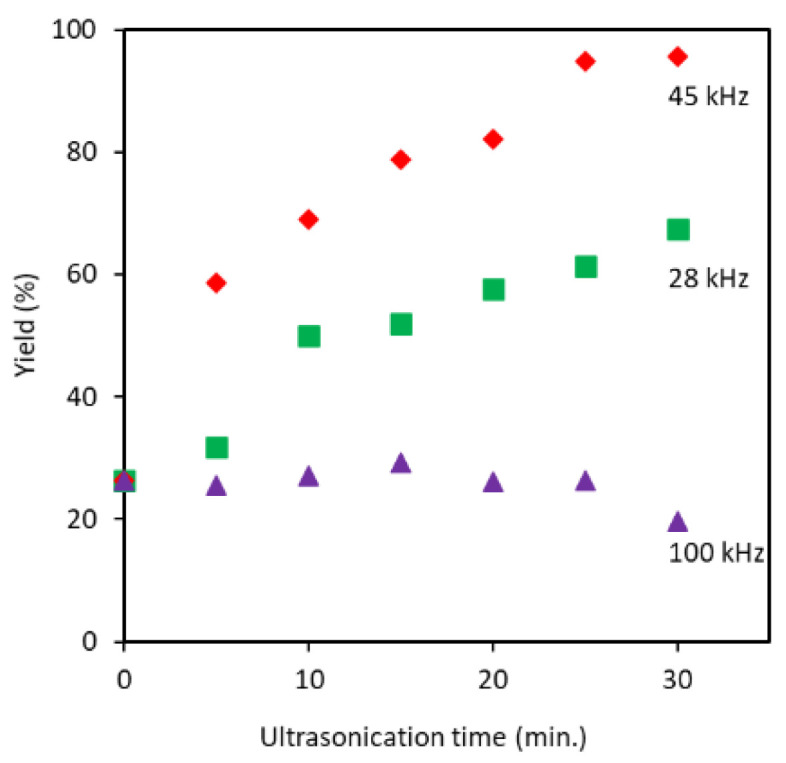
Dependence of yields on the ultrasonication frequency at 28, 45, and 100 kHz and ultrasonication time.

The disintegration efficiency was strongly dependent on ultrasonication frequency and treatment time. A higher yield was registered for 45 kHz followed by 28 kHz, as shown in Figure 1. When the ultrasonication minute was increased to 30, proportionally the yield was raised to 96%, in the case of 45 kHz. At a frequency of 28 kHz, the maximum yield obtained was 67% in 30 min of processing. A further increasing of frequency to 100 kHz did not increase yield within 30 min of processing at all. The observed trend can be explained based on the effect of cavitation bubble dynamics on the individualization process. Frequency controls cavitation bubble size, the number of cavitation bubbles, and the implosion energy. The energy generated from the cavitation bubble increases with bubble size and with a decrease in ultrasonic frequency [18]. Bigger cavitation bubbles are produced at lower frequency or higher wavelength since the bubble experiences enough time of rarefaction to grow before they implode [22]. On the other hand, at higher frequency a large number of bubbles with weak impact are created [19,23]. Therefore, at a frequency of 28 kHz, larger size and smaller number cavitation bubbles with strong implosion energy are created. However, in the case of 100 kHz, relatively higher number of bubbles and smaller size with weak impacts are created. Therefore, the optimum bubble size, number, and impact for efficiently crushing the partially deacetylated chitin are created at 45 kHz. Thus, we performed a characteristic analysis of partially deacetylated chitin crushed by ultrasonic waves at a frequency of 45 kHz. On the other hand, when the original chitin was used for comparison, chitin could not be dispersed in water even after 30 min of ultrasonic treatment at a frequency of 45 kHz. This suggests that a deacetylation pretreatment is required for efficient disintegration of chitin.

### 3.2. Morphological and Structure Characterization of Partially Deacetylated Chitin Nanofibers

**Figure 2:**
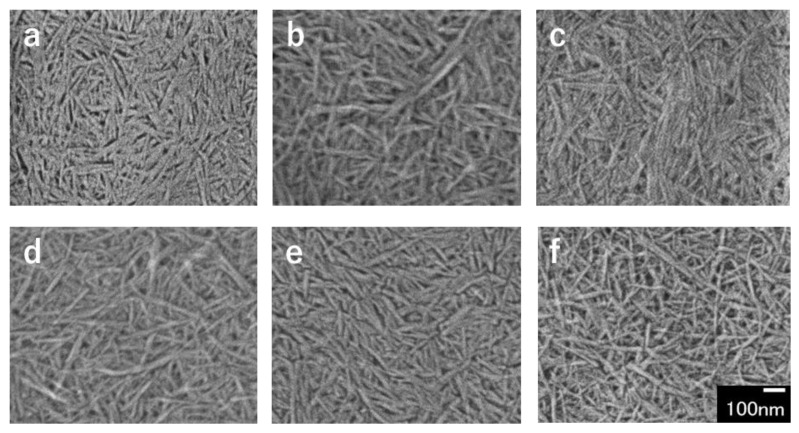
FE-SEM images of partially deacetylated chitin nanofibers with ultrasonication time of (**a**) 5, (**b**) 10, (**c**) 15, (**d**) 20, (**e**) 25, and (**f**) 30 min.

FE-SEM images of partially deacetylated chitin ultrasonicated at a frequency of 45 kHz, which is the optimum frequency in yield are shown in Figure 2. The disintegrated product obtained by ultrasonication was a spindle-shaped nanofiber with a width of about 10 nm and a length of several hundred nm. The nanofibers appeared to be uniform in width and length and to be smaller in width and length depending on the ultrasonic processing time, which will be described in detail later.

**Figure 3:**
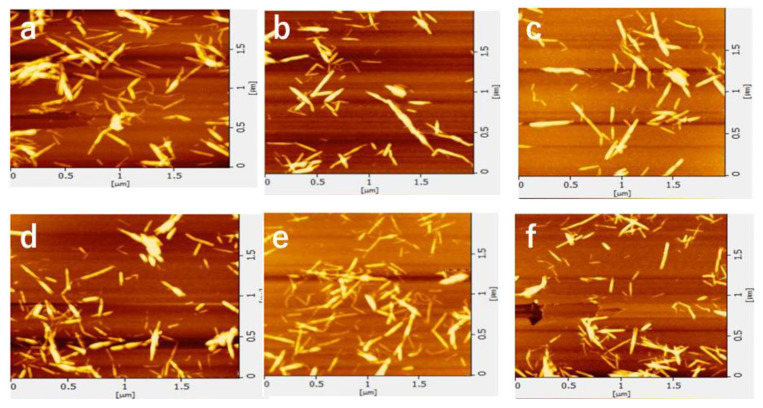
AFM images of partially deacetylated chitin nanofibers with ultrasonication time of (**a**) 5, (**b**) 10, (**c**) 15, (**d**) 20, (**e**) 25, and (**f**) 30 min.

**Figure 4:**
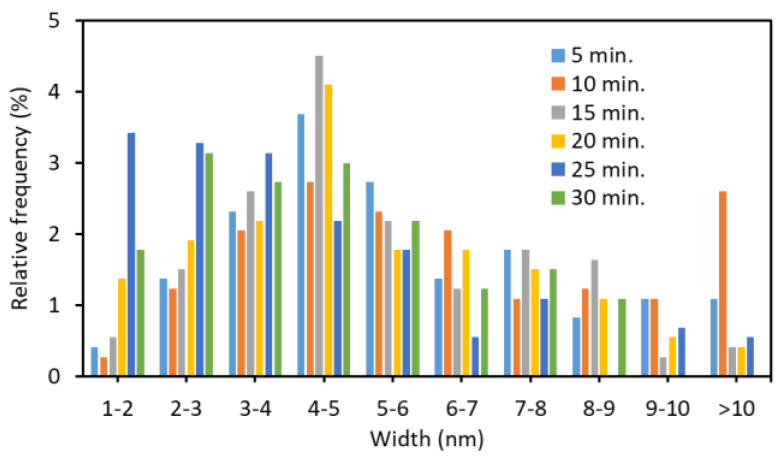
The effect of ultrasonication time at 45 kHz on the distribution of partially deacetylated chitin nanofiber.

The AFM images of the nanofibers and the width distribution are shown in Figure 3 and Figure 4, respectively. The shape of the nanofibers observed by AFM was in good agreement with the image by FE-SEM. The ultrasonication process at 45 kHz appears to reduce the width and length of the nanofiber. When the ultrasonic treatment time was 10 min or less, some nanofibers had a width of 10 nm or more. As the processing time increased, the width became smaller (from 4 to 5 nm was the most). Additionally, the fiber width distribution became narrower. This is because nanofibers are being disintegrated by ultrasonic treatment.

**Figure 5:**
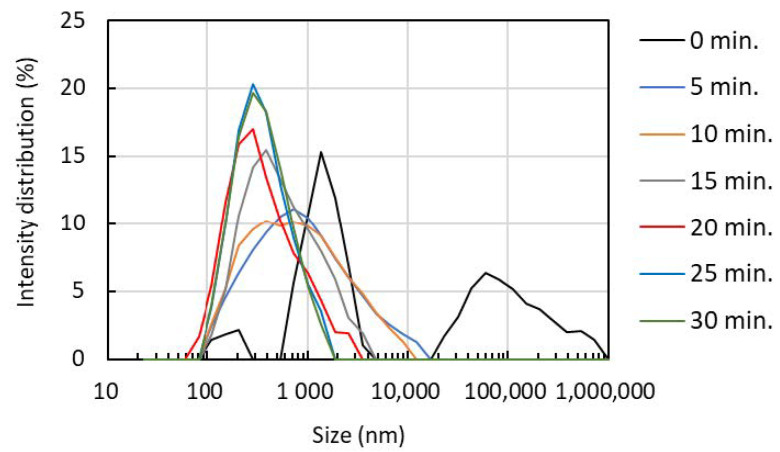
The effect of ultrasonication time on partially deacetylated chitin nanofiber size distribution.

The hydrodynamic diameter and its distribution were obtained by the dynamic light scattering method (Figure 5). The nanofiber rotates in water by Brownian motion, and the particle size correlates with the length of the nanofiber. We also evaluated the degree of dispersion, which is an index of uniformity from the particle size distribution. When the ultrasonic treatment time was 0 min, coarse particles having a particle diameter of 10,000 nm or more were observed. However, after ultrasonication for 5 min, the coarse particles were completely disintegrated into fine nanofibers. When the treatment time was increased from 5 min to 15 min and 20 min, the disintegration further progressed, the particle size became smaller, and the distribution became narrower. Table 1 shows mean average size, and crystallinity index of partially deacetylated α-chitin nanofiber. When the treatment time was 0 min, the average particle size was 2025 nm but decreased to 629 nm after 5 min of treatment. By increasing the sonication time, the average particle size decreased. However, after the treatment for 20 min it almost leveled off. The polydispersity index was 0.78 when the sonication time was 0 min, but significantly reduced to 0.35 after 5 min treatment. The polydispersity index decreased slightly as the sonication time increased. Consequently, ultrasonication at a frequency of 45 kHz converts partially deacetylated chitin into nanofibers. The length and width changed depending on the processing time. The morphology of chitin nanofibers when sonicated at a frequency of 45 kHz for more than 20 min shows good agreement with those reported in the past [15].

**Figure 6:**
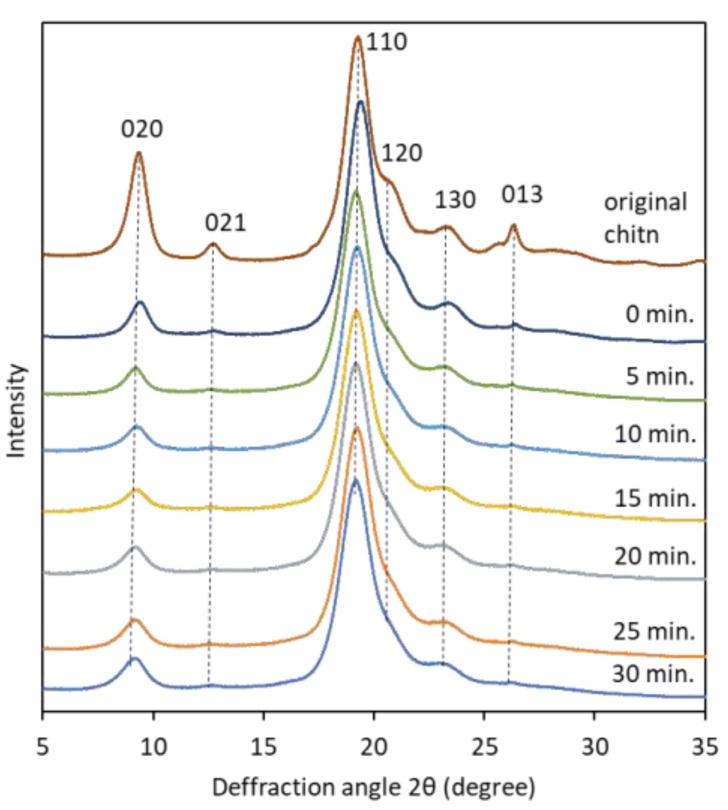
X-ray diffraction patterns of pure α-chitin powder and partially deacetylated α-chitin with different ultrasonication time.

The X-ray diffraction profiles of pure chitin, partially deacetylated chitin, and nanofiber with respective ultrasonication minute was used to investigate change in crystallinity (Figure 6). The diffraction profile of α-chitin in the range of 5° ≤ 2*θ* ≤ 35° shows more intense peaks located at 2*θ* = 9.36° and 19.28° which corresponds to (020) and (110) crystallographic plane. Less intense peaks can be observed at 2*θ* = 12.80°, 20.91°, 23.22°, and 26.39° which associated with (021), (120), (130) and (013), respectively, and close to previous reports [24,25]. Relative crystalline index was determined from intense peak of 110 plane in the range 19° ≤ 2*θ* ≤ 20° and the amorphous diffraction at 16° [21]. As shown in Table 1, the relative crystallinity index of partially deacetylated chitin nanofibers treated with ultrasonic waves was about 87% regardless of the treatment time, which was almost constant. This suggests that sonication does not damage the crystalline region of chitin, (that is, it breaks the amorphous region). This result is consistent with previously reported studies [15]. In addition, the FT-IR spectrum of the partially deacetylated chitin nanofiber did not change depending on the processing time. This suggests that sonication does not alter the chemical structure of chitin (Figure S1).

**Figure 7:**
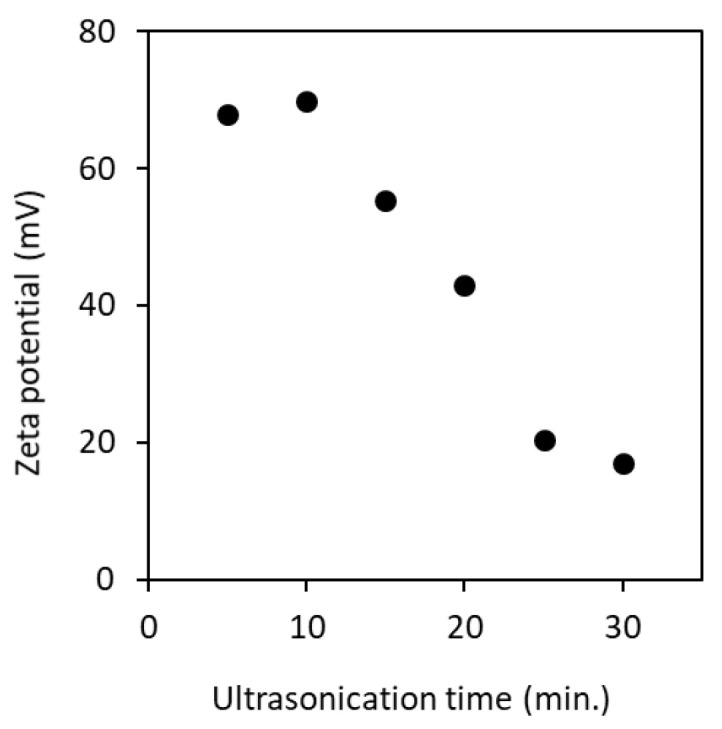
Zeta potential of partially deacetylated chitin nanofiber as a function of ultrasonication time.

ζ-Potential of the partially deacetylated chitin nanofiber aqueous dispersion was measured. Figure 7 shows the ζ-potential when ultrasonicated for 5 to 30 min. Nanofibers had a cationic surface charge under acidic conditions. This is due to the protonation of the amino group (–NH_3_^+^) on the surface of the nanofiber. Thus, pH of the dispersion has a strong effect on the ζ-potential [15]. The electrostatic repulsive force between nanofibers with a cationic surface charge contributes to the dispersion stability in water. The ζ-potential of the nanofibers sonicated for 5 min had a high positive surface charge (+68 mV), and this value was decreased with increasing the processing time. It is suggested that the surface area of the nanofiber is increased by the ultrasonic treatment and, as a result, the ionic strength is increased. At higher ionic strength, the shielding effect becomes strong. This shielding might have led to reduction in effective charge of the cations, so that there was a shrink in the slipping plane. That is, the higher cationic surface charge offsets the electrostatic repulsive force of the nanofiber in water. This may reduce the Debye length in addition to affecting shrink in slipping plane, resulting in an increase in Van der Waals force. According to the Derjaguin–Landau–Verwey–Overbeek (DLVO) theory, the total interaction energy is considered to be a combination of Van der Waals and electrostatic forces [26,27]. As the sonication time increased, the Van der Waals force increased and the electrostatic force decreased, which suggests the decrease of ζ-potential. The effect of decrease in ζ-potential was clearly shown on the viscosity of nanofibers as described below.

### 3.3. Properties of Partially Deacetylated Chitin Nanofiber Dispersion in Water

**Figure 8:**
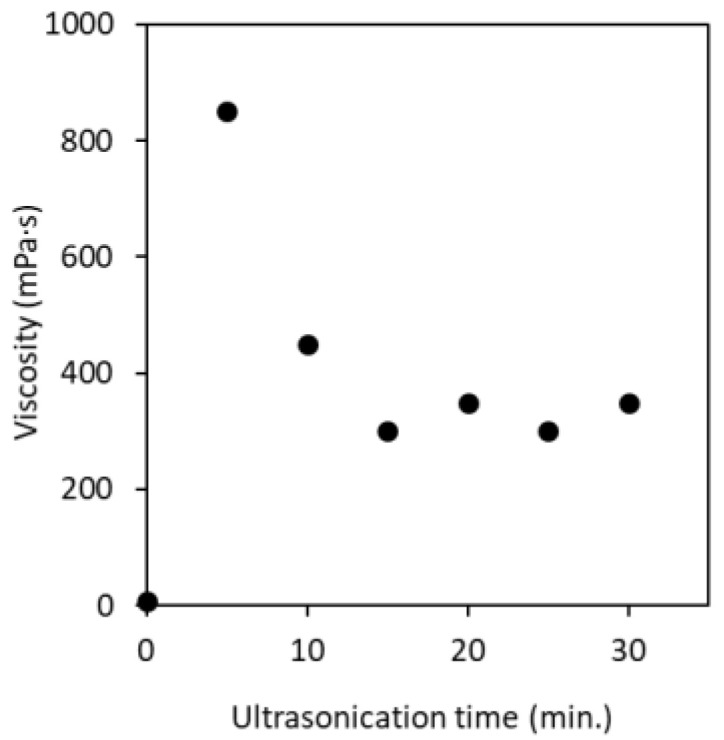
Viscosity of partially deacetylated chitin nanofiber suspension as a function of ultrasonication time.

The viscosity of partially deacetylated α-chitin nanofiber at a solid content of 0.60 wt% with different ultrasonication time is shown in Figure 8. Before ultrasonication, the viscosity of partially deacetylated chitin was relatively low (9.25 mPa·s). This is due to the fact that, before the ultrasonic treatment, the unindividualized partially deacetylated chitin fibers are thick and have a wide distribution, so that there is little entanglement between the fibers. After 5 min of sonication, the viscosity increased significantly, reaching 850 mPa·s. On the other hand, after 10 min or more of treatment, the viscosity decreased and became almost constant in 15 min. This is related to the morphology of the nanofibers. That is, chitin was converted into nanofibers by sonication and interacts with each other in water to increase its viscosity. On the other hand, sonication for 10 min or more shortens the fiber length. Shortening the nanofibers suppresses the entanglement of the nanofibers, resulting in a decrease in viscosity. The ζ-potential of nanofibers is also involved. That is, the fiber length decreases with the ultrasonic treatment. As disintegration progresses, new interface of chitin was appeared. Then, the ionic strength increases and the ζ-potential decreases as described above. A decrease in ζ-potential reduces the interaction and dispersibility of nanofibers, resulting in a decrease in viscosity. Moreover, the decrease in ζ-potential reduces the electrostatic repulsive force of the chitin nanofibers, and then reduces the viscosity of the dispersion. From the above, it was found that the viscosity of the nanofiber can be controlled by the ultrasonic treatment time. The decrease in viscosity makes it possible to increase the concentration of nanofibers, which is industrially advantageous.

**Figure 9:**
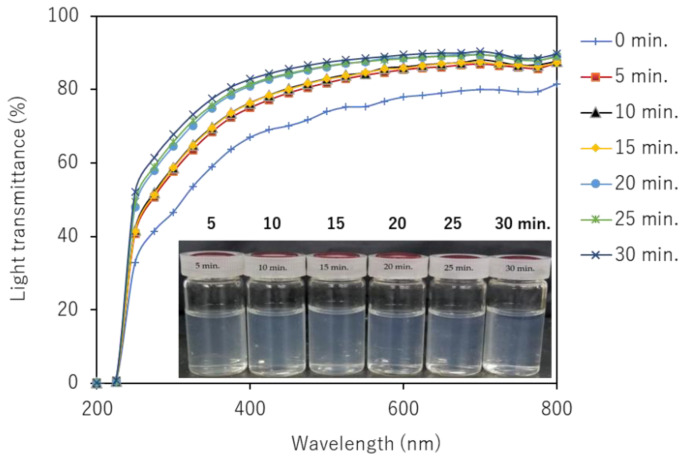
Photographs and UV-Vis transmittance spectra of partially deacetylated chitin nanofiber with respect to ultrasonication time and those photographs.

The UV-Vis transmittances of partially deacetylated chitin nanofiber with 0.1 wt% dispersion at different ultrasonication time were shown in Figure 9. The transmittance of the unsonicated partially deacetylated chitin dispersion was 78% at 600 nm, but the transmittance increased as the ultrasonic treatment time increased. This is clearly due to the nanofiber morphology. That is, sonication reduces the width and length of nanofibers and narrows their distribution. Such a change in morphology suppresses light scattering of nanofibers and improves transparency. In addition, the positive charge on the surface of the nanofiber promotes stable dispersion for a long period of time, enabling individual dispersion.

## 4. Conclusions

Partially deacetylated chitin could be disintegrated to the nano level using a commercially available ultrasonic cleaner. The positive charge of partially deacetylated chitin facilitates downsizing, also allows for a high dispersibility. The highest yield of ultrasonic waves was achieved at 45 kHz, reaching 96% after 30 min of treatment. On the other hand, at 100 kHz, it could not be downsized at all. The sonicated chitin was a nanofiber individualized and homogeneously dispersed in water. The fiber width and length can be controlled by changing the processing time. The fiber width of the nanofiber treated for 30 min was about 4–5 nm, and the average fiber length was 294 nm. Therefore, the aspect ratio was estimated to be about 60–70. Ultrasonic cleaners can be purchased inexpensively and consume less power. Partially deacetylated chitin nanofibers have excellent physiological functions. The results of this study will lead to clinical research on partially deacetylated chitin nanofibers and promotion of their use as pharmaceuticals and medical devices.

## Figures and Tables

**Table 1:** Mean average size, polydispersity index, and crystallinity index of partially deacetylated α-chitin nanofiber.

Ultrasonication Time (min)	Mean Average Size (nm)	Polydispersity Index	Crystallinity Index (%)
α-chitin powder	-	-	89.88
0	2025	0.78	89.73
5	629	0.35	87.02
10	549	0.35	86.31
15	429	0.29	85.37
20	310	0.32	87.87
25	309	0.29	87.05
30	294	0.26	87.05

## Data Availability

Data are contained within the article.

## Supplementary Materials

The following are available online at https://www.mdpi.com/article/10.3390/polym13152501/s1. Figure S1: FT-IR spectra of pure α-chitin powder (a), partially deacetylated α-chitin (b), and ultrasonicated for 5 (c), 10 (d), 15 (e), 20 (f), 25 (g), and 30 min (h).
