# Supplementary material for: Optimum Preparation Conditions for Highly Individualized Chitin Nanofibers Using Ultrasonic Generator
Source: Polymers (Basel). 2021 Jul 29;13(15):2501. doi: 10.3390/polym13152501 (PMC8348391; doi:10.3390/polym13152501)
Supplement: Supplementary file 1 [file polymers-13-02501-s001.zip › polymers-1300507-supplementary.pdf]

Article

Optimum Preparation Conditions for Highly Individualized Chitin Nanofibers Using Ultrasonic Generator

Dagmawi Abebe Zewude [1], Hironori Izawa, [1,2] and Shinsuke Ifuku [1,2,*]

[1] Graduate School of Engineering, Tottori University, 4-101 Koyama-Minami, Tottori 680-8550, Japan; d19t3106x@edu.tottori-u.ac.jp (D.A.Z.); h-izawa@tottori-u.ac.jp (H.I.)
[2] Center for Research on Green Sustainable Chemistry, Tottori University, Tottori 680-8550, Japan
* Correspondence: sifuku@tottori-u.ac.jp

Supplementary Materials

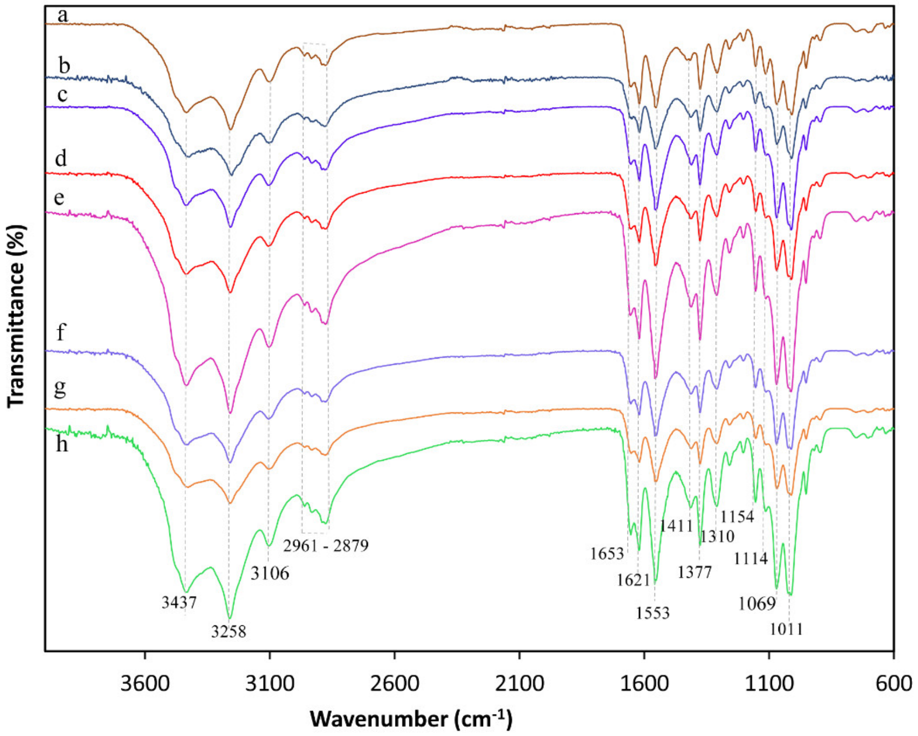

Figure S1. FT−IR spectra of pure α-chitin powder (a), partially deacetylated α-chitin (b), and ultrasonicated for 5 (c), 10 (d), 15 (e), 20 (f), 25 (g), and 30 min (h).
